# Biomimetic Surface Engineering to Modulate the Coffee-Ring Effect for Amyloid-β Detection in Rat Brains

**DOI:** 10.3390/biomimetics8080581

**Published:** 2023-12-01

**Authors:** Changxin Wang, Lei Li, Jiaze Li, Jun Zhang, Zhi-Bei Qu

**Affiliations:** Department of Medicinal Chemistry, School of Pharmacy, Fudan University, 826 Zhangheng Road, Shanghai 201203, China; 23211030090@m.fudan.edu.cn (C.W.); 21301030041@m.fudan.edu.cn (L.L.);

**Keywords:** coffee-ring effect, biosensor, amyloid β, gold nanoparticles, brain ischaemia

## Abstract

Surface engineering of nanoparticles has been widely used in biosensing and assays, where sensitivity was mainly limited by plasmonic colour change or electrochemical responses. Here, we report a novel biomimetic sensing strategy involving protein-modified gold nanoparticles (AuNPs), where the modulation strategy was inspired by gastropods in inhibition of coffee-ring effects in their trail-followings. The so-called coffee-ring effect presents the molecular behaviour of AuNPs to a macroscopic ring through aggregation, and thus greatly improves sensitivity. The assay relies upon the different assembly patterns of AuNPs against analytes, resulting in the formation or suppression of coffee-ring effects by the different surface engineering of AuNPs by proteins and peptides. The mechanism of the coffee-ring formation process is examined through experimental characterizations and computational simulations. A practical coffee-ring effect assay is developed for a proof-of-concept target, amyloid β (1–42), which is a typical biomarker of Alzheimer’s disease. A novel quasi-titrimetric protocol is constructed for quantitative determination of the target molecule. The assay shows excellent selectivity and sensitivity for the amyloid β monomer, with a low detection limit of 20 pM. Combined with a fluorescent staining technique, the assay is designed as a smart sensor for amyloid β detection and fibrillation evaluation in rat cerebrospinal fluids, which is a potential point-of-care test for Alzheimer’s disease. Connections between amyloid fibrillation and different courses of brain ischaemia are also studied, with improved sensitivity, lower sample volumes that are required, convenience for rapid detection, and point-of-care testing.

## 1. Introduction

When a drop of a solution dries on a flat surface, a ring-like stain is deposited at the original drop edge. This so-called coffee-ring effect is ubiquitous but has not been thoroughly investigated [1]. This phenomenon is influenced by the particle shape [2] and size [3] of particles in the solution, by their surface modification, and by their hydrophilicity [4]. The coffee-ring effect involves a complex but robust dynamic ensemble and remains a novel and exciting area for researchers [5,6] because it connects phenomena from the nanoscale to the macroscale. The testing of the coffee-ring effect only requires a piece of hydrophilic substrate (e.g., glass, wood, or paper) and a small droplet of solution. The residue of the dried solution can be identified by the naked eye and requires no complicated instrument. Thus, the coffee-ring effect is convenient to serve as a facile strategy in analytical chemistry for sensitive diagnostic applications [7,8], especially for high-throughput detection in point-of-care platforms [9].

In nature, snails and slugs leave a trail-following from moving on a layer of costly mucus. The silver trails were demonstrated to exhibit multiple functions, including the release of water- or airborne pheromones for mate searching. Some snails prefer to move over previously laid mucus trails to save energy and reduce production of their own mucus [10]. For either function, the formation of the coffee-ring effect in trail-followings should be inhibited. Gastropods inhibit the coffee-ring effect in their mucus by secreting mucins and other protein–carbohydrate complexes, which help to increase the affinities between particles in mucus. The successful strategy of snails in inhibiting coffee-ring effects have been long used by engineers, as well as painters in the painting of dedicated patterns [6].

Learning from snails, we proposed a coffee-ring effect-based strategy to construct a biomimetic optical sensor using gold nanoparticles (AuNPs), the most popular nanomaterial, utilized across a wide range of scientific research fields, including serving as the building blocks of biomimetic structures [11]. We designed the sensor through modulating coffee-ring effects by surface engineering of AuNPs, where stronger AuNP-AuNP interactions lead to the suppression of coffee-ring strains. Owing to their unique surface plasmon resonance (SPR) properties, AuNPs display size- and distance-dependent optical absorption [12] so that colorimetric assays are the most common use of AuNPs because of their high sensitivity, ease of construction, and suitability for naked-eye detection [13]. However, the applications of SPR-based AuNP colorimetric assays is often limited. This is because colour changes in colloidal AuNP solutions can be easily triggered by high ionic strength, protein absorption, or desorption of functionalized molecules; these conditions may therefore result in false-positive signals [14]. Moreover, an evident colour change in colloidal AuNP solutions only occurs when the AuNPs aggregate at high levels. The colour of the AuNP colorimetric assays typically shifts from red to blue (or purple) or vice versa. This colour change, however, does not often offer a distinguishable contrast by the naked human eye. Thus, there is a demand to improve AuNP colorimetric assays, especially their sensitivity and robustness. A coffee-ring effect-based sensor utilizes the tuneable aggregation properties of modified gold nanoparticles by surface engineering in response of target molecules. In our work, the absorbance of the coffee-ring effect is a more sensitive sensor than the absorbance of the solution. The well-dispersed AuNPs in a droplet of colloidal AuNP solution dry to form a distinct ring-fashion stain, whereas the remainder of the solution does not exhibit such a residue, even when the AuNPs slightly aggregate without an obvious colour change. This coffee-ring-effect-based platform was successfully applied for the sensitive and selective identification of the amyloid β (Aβ) monomer, which is a well-known biomarker of Alzheimer’s disease [15]. A technique to evaluate the fibrillation level of Aβ was further developed, using fluorescent staining. A smart sensor that can simultaneously detect the concentration of Aβ and evaluate the fibrillation level was developed. Furthermore, the delicate balance between the Aβ concentration and its fibrillation level (in rat cerebrospinal fluids) on brain ischaemia was explored. This coffee-ring-effect-based sensing technique using colloidal AuNPs appears promising for potential analytical chemistry and diagnostic applications.

## 2. Materials and Methods

### 2.1. Synthesis of Gelsolin-Modified AuNPs (Gel-AuNPs)

AuNPs of 20 nm average diameter were fabricated as previously reported. Gel-AuNPs were fabricated via a one-pot modification method. Briefly, 10 mM gelsolin was added to 5.0 mL of a colloidal AuNP solution and stored at room temperature with gentle stirring for 5 h. Then, bovine serum albumin (BSA) was used to block the nonspecific binding sites of the AuNPs. The obtained modified nanoparticles were separated with centrifugation (5000 rpm) and were washed three times using ultrapure water. 

### 2.2. Design of the Coffee-Ring-Effect-Based Assays

Glass slides were carefully washed with *aqua regia*, alcohol, and ultrapure water. For the Aβ monomer determination, droplets of the Gel-AuNPs were dropped onto a glass slide with a microinjector (10 mL, Eppendorf, Hamburg, Germany). The glass slide was then stored in steady air under ambient conditions until the liquid was completely evaporated. The humidity was controlled below 30%. Then, the deposit on the slides were dipped into ultrapure water to remove any precipitated salt crystals before observation by naked eye, by a portable sensing platform with a smartphone, and by optical microscopy. For the Aβ fibrillation analysis, the glass slide was dipped into a 10 mM thioflavin T (ThT) solution for the fluorescent staining of the Aβ fibrils.

### 2.3. Animal Experiments

All procedures involving animals were conducted with the approval of the Animal Ethics Committee in Fudan University, China. Male SD rats (200–250 g) were purchased from Shanghai SLAC Laboratory Animal Co., Ltd. (Shanghai, China), and acclimatized for a week. In detail, the SD rats were randomly divided into three groups. The rats were anaesthetized with chloral hydrate (300 mg/kg, i.p.) and additional injections were performed as needed to maintain anaesthesia. Surgeries were carried out to introduce intraluminal sutures into the external carotid artery (ECA) to induce brain ischaemia. After the surgery, the rats were placed in a warm incubator whilst recovering from the anaesthesia. For the long-term ischaemia group (*n* = 5), the cerebrospinal fluid was collected after 30 days. For the short-term ischaemia group (*n* = 5), the cerebrospinal fluid was collected after 3 days. The remaining rats (normal group, *n* = 5) were housed without any further treatment. The rats were then anaesthetized with ether, their cerebrospinal fluid was collected and the Aβ concentration, and fibrillation analyses was carried out before sacrifice.

## 3. Results

### 3.1. Design of the Coffee-Ring-Effect-Based Sensing Platform

The coffee-ring effect is dependent on the size and the shape of the particle, as well as the stickiness of the surfactant functionalization. Therefore, we assumed that the coffee-ring effect would be eliminated when the AuNPs aggregated. We used negatively charged AuNPs to verify our assumption. Different volumes of NaCl or HCl solutions were added into the AuNP solution to induce aggregation. Then, droplets of well-dispersed AuNPs and AuNPs at different aggregation levels were deposited onto a glass slide. Subsequently, the drops were allowed to evaporate, and the dried suspensions’ deposits were studied. The coffee-ring effect was not observed when the AuNPs underwent slight aggregation.

For well-dispersed AuNPs, the coffee-ring effect was driven by capillary fluid flow during the evaporation process. However, when the AuNPs began to aggregate, anisotropic structures of the AuNPs such as dimers and chains of AuNPs emerged in the solution as the attraction force between the AuNPs become stronger. This anisotropy and stronger affinity between the AuNPs prevented the capillary fluid flow within the solution bulk and at the air–fluid interface. At higher aggregation levels, highly densified AuNPs assembled into micrometre-sized plaques. These dense plaques were too heavy to be carried away by the capillary fluid flow [16]. Thus, these phenomena prevented the coffee-ring effect.

**Figure 1:**
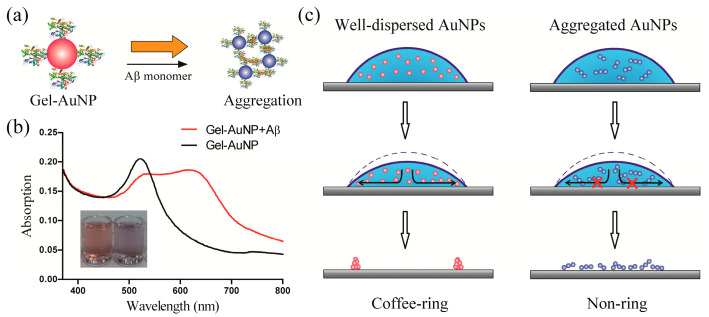
Design of the coffee-ring effect based optical sensor. (**a**) Schematic illustration for the recognition of gelsolin-functionalized AuNPs (Gel-AuNPs) and the Aβ monomer. (**b**) UV-vis absorption spectra and images (inset) of Gel-AuNPs in the presence and absence of the Aβ monomer. (**c**) Schematic representation of the coffee-ring effect of the AuNPs and its prevention by aggregation.

Based on these promising results, we further expanded this strategy to the development of a sensitive optical sensing system (Figure 1). A selective biosensor for the Aβ monomer was designed based on the visualization of the coffee-ring effect. Gelsolin, a secretory protein that selectively binds to Aβ by a recognition behaviour similar to that between an antibody and antigen, was immobilized onto AuNPs via a one-pot method (Gel-AuNPs, see Methods). The advantage of the modifying protein gelsolin is reflected in two aspects. First, the involvement of gelsolin was largely inspired by the gastropods in the inhibition of coffee-ring effects in their trail-followings, where sticky proteins play essential roles. Gelsolin with an isoelectric point of 6.1 was negatively charged in working buffer, such that the interactions between gelsolin were dominated by electrostatic repulsions. In the presence of amyloid beta, however, strong affinity occurred between gelsolin and amyloid peptides, by forming sticky protein–peptide complexes. Second, gelsolin was known to exhibit selective affinity to amyloid beta peptides, which is essential to the construction of selective assay for amyloid beta in rat brain. Transmission electron microscopy (TEM) was used to validate the successful fabrication of Gel-AuNPs (Figure S4). It was demonstrated that after gelsolin modification, the morphology of AuNPs did not change much. High-resolution (HR) TEM images showed very clear Au (111) lattices with a distance of 0.3 nm.

In our previous work, gelsolin was shown to selectively bind to Aβ monomers at more than one recognition site [17,18]. Gel-AuNPs were observed to aggregate with each other in the presence of Aβ monomers. The aggregation of Gel-AuNPs in the presence of Aβ resulted in the suppression of the coffee-ring effect when the solution was deposited onto a glass substrate. The level of suppression of the coffee-ring effect was very sensitive to the aggregation level. In the absence of Aβ, the dried Gel-AuNP droplet left a well-defined ring. However, in the presence of Aβ, there was no ring shape after the droplet was dried. At this concentration, the addition of Aβ did not result in an obvious colour change. The Gel-AuNPs showed excellent specificity for the Aβ monomer against other competing agonists, including Aβ oligomers, Aβ fibrils, nucleic acids, amino acids, and proteins. This protocol can be viewed as a qualitative analytical method of the Aβ monomer.

**Figure 2:**
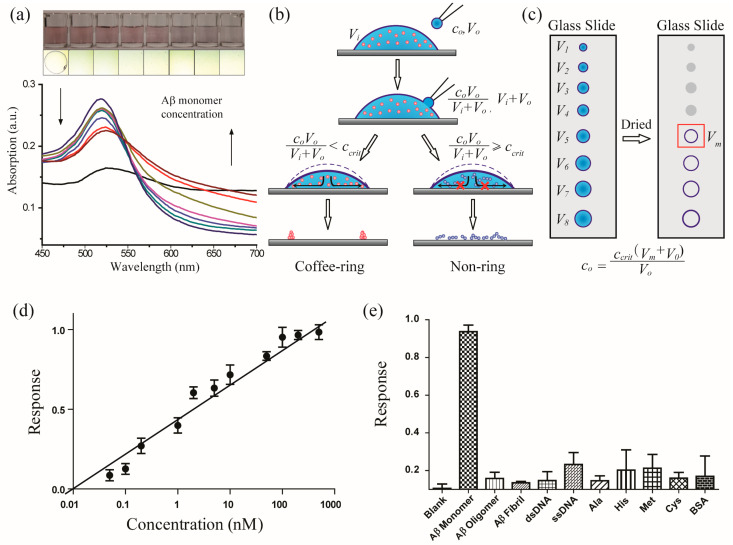
Quantification using the coffee-ring-effect-based optical sensor. (**a**) Images of the solutions and dried deposits on a glass slide of the Gel-AuNPs with various levels of aggregation induced by different concentrations of Aβ (from 0.1 nM to 5 μM). (**b**) Schematic illustration of the protocol using for the quantitative Aβ detection with Gel-AuNPs. (**c**) Determining the minimum volume of Gel-AuNPs (*V_m_*) before the coffee-ring effect is suppressed. (**d**) Linear plot of the coffee-ring effect optical sensor versus different concentrations of Aβ. (**e**) Selectivity of the coffee-ring effect assay against various competing compounds. The concentration of Aβ monomer was 100 nM, while Aβ oligomer and fibril was 5 μM, nucleic acids and BSA were 10 μM, and amino acids were 100 μM in concentration.

Quantification is essential in analytical chemistry and diagnostic detection. The concentration of Aβ in brain tissues has been correlated with Alzheimer’s disease. Several groups reported relatively low concentrations of the Aβ monomer in the cerebrospinal fluids of Alzheimer’s disease patients [19]. Thus, a quantitative analysis of Aβ is necessary [20,21,22,23]. Researchers have utilized the coffee-ring effect to construct assays, e.g., Wong and coworkers have suggested a chromatography-like behaviour of the coffee-ring effect and proposed its application in separations and fluorescent detections; Chen and coworkers developed a method for detecting DNA using a hybridization-induced suppression of the coffee-ring effect [24]. However, these techniques were not capable of performing quantitative analyses. Indeed, to the best of our knowledge, an accurate, quantitative analysis of the coffee-ring effect has never been achieved. In this work, a novel quantitative method was proposed to overcome the limitations associated with coffee-ring-effect-based sensor (Figure 2).

A quasi-titrimetric method was introduced for the quantification. The critical concentration (*c_crit_*) is the minimum concentration of Aβ that can suppress the formation of a coffee-ring of Gel-AuNPs. The critical concentration of this system can be adjusted by varying the concentration of Gel-AuNPs and by the degree of gelsolin modification. The *c_crit_* can be determined using an external reference. The quantitative analysis was performed by the following quasi-titrimetric protocol. First, different volumes of Gel-AuNPs (V_i_) were dropped onto glass slide substrates. Then, Aβ aliquots of volume *V*_0_ and concentration *c*_0_, were fused with each drop of Gel-AuNPs. Finally, the droplets were slowly evaporated and the deposit residue of each droplet was observed. This method was used to determine the minimum volume of Gel-AuNPs (*V_m_*) for a constant quantity of Aβ before the coffee-ring effect was suppressed. 

The concentration *c*_0_ of Aβ used in the titration was calculated as follows:

(1) $$c_{0}=\frac{c_{crit}\left(V_{m}+V_{0}\right)}{V_{0}}$$

The sensitivity of the coffee-ring-effect-based optical sensor was very high. The limit of detection (LOD) for Aβ was as low as 20 pM, which is remarkably lower than that of the Gel-AuNP-based colorimetric channel (0.5 nM) in this work (see Supporting Information). Various compounds, including Aβ oligomers, Aβ fibrils, proteins, nucleic acids and amino acids, were tested to verify the selectivity of the assay. None of the above-mentioned compounds showed similar suppression of the coffee-ring effect and would not affect the quantitative determination of Aβ monomer in artificial cerebrospinal fluids (aCSF) (Figure 2e). We performed a comparison analysis of the coffee-ring effect assay with conventional AuNP colorimetric methods in Table S1. It can be concluded that the coffee-ring-based assay required a much lower volume of the sample and remarkably higher sensitivity than the conventional colorimetric method. For selectivity and measurement time, they showed comparative performance. But it should be noted that the conventional colorimetric method showed better reproducibility than the coffee-ring effect assay, which should be improved in the future.

### 3.2. Mechanism of the Coffee-Ring Effect of AuNPs

We next examined the mechanism responsible for the sensitivity of the coffee-ring effect to aggregation. We hypothesized that three major factors contributed to the sensitivity of the coffee-ring-effect-based sensor. The first is associated with the nature of the coffee-ring effect. During the evaporation process, AuNPs are highly concentrated at the edge of the drop. Nonvisible nanoparticles are visualized at a macroscopic scale. Generally, the area of the coffee ring occupies approximately 8% of the total area of the droplet. The high concentration of AuNPs in this very small area largely enhances the sensitivity for indicating the aggregation level of the nanoparticles. Second, AuNPs aggregate into chain-like assemblies [25], which play an essential role in the suppression of the coffee-ring effect. The formation of chain-like assemblies drives AuNPs to anisotropic structures with much stronger interparticle affinity. The existence of anisotropic structures in solution significantly resists the radial outward flow, the driving force and source of the coffee-ring effect [26]. It has been demonstrated that a small quantity of anisotropic chain-like assemblies of AuNPs can readily damage the coffee-ring. Moreover, as the liquid droplet volume decreases, the concentration of the detected molecule increases, i.e., the non-volatile Aβ monomer is largely enriched before the coffee-ring pattern begins to emerge. The enrichment of the detected molecules can be two orders of magnitude or higher in the coffee-ring-effect-based optical sensors, and thus contributes towards the improved sensitivity.

**Figure 3:**
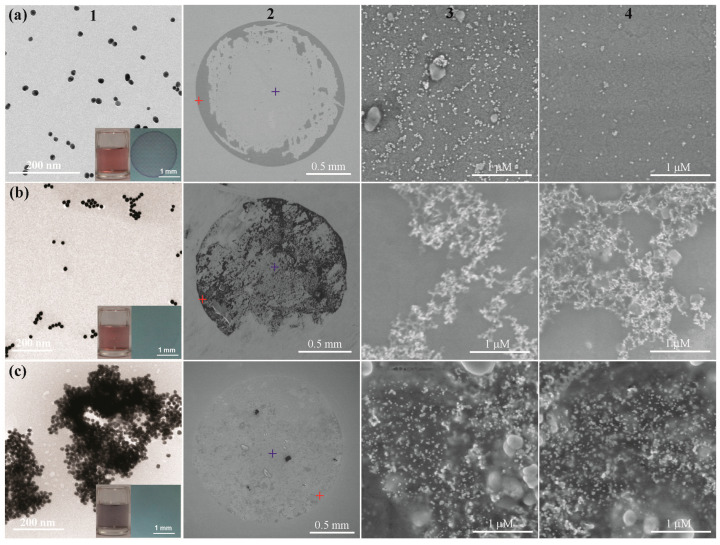
TEM (column 1) and SEM (column 2) images for the entire deposit residue, at the deposition edge (column 3) and at the deposition centre (column 4) of the Gel-AuNPs in the absence of Aβ (**a**), and in the presence of 1 nM Aβ (**b**) and 0.5 μM Aβ (**c**). Red cross denoted the zoomed-in SEM images of regions for the droplet deposition edges, while blue cross denoted the ones for regions for the droplet deposition centres. Insets: images of the solutions and the deposit formed after evaporation on a glass slide.

Transition electron microscopy (TEM) and scanning electron microscopy (SEM) (Figure 3) were used to examine the aggregation mechanism. The well-dispersed AuNPs deposited more densely at the edge than those in the centre. No obvious aggregation was observed in both areas. At low aggregation levels, the AuNPs aggregated into chain-like structures. As shown in Figure 3b, branched chain structures of AuNPs were found at the edge and at the centre of the deposit. Furthermore, at high aggregation levels, large plaques of highly dense AuNPs were observed throughout the deposit. We further utilized Monte Carlo simulations to support our proposed mechanism (see Supplementary Information). The deposition process of colloidal AuNPs can be divided into two stages. In the first stage, i.e., the edge deposition process (EDP), the AuNPs predominantly deposited at the edge of the droplet. In the second stage, i.e., the planar deposition process (PDP), the liquid phase became very thin such that the deposition occurred along the entire air–liquid interface. The formation of the coffee ring is determined by the stage that dominated during the drying process.

### 3.3. Animal Models and Real Sample Detection

**Figure 4:**
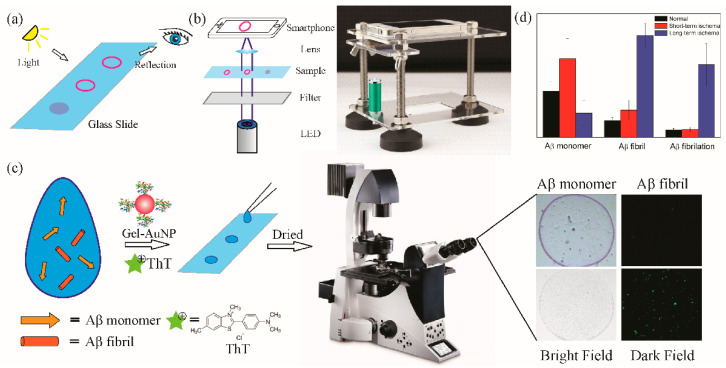
Detection modes of coffee-ring effect optical sensors using AuNPs. (**a**) Primary detection by the naked eye. (**b**) Portable detection platform for point-of-care applications using a smartphone. (**c**) Fluorescent staining with ThT and the application to evaluate the Aβ fibrillation levels using fluorescent microscopy. (**d**) Results of the Aβ monomer, fibril concentrations, and fibrillation levels in long-term and short-term brain ischaemia in comparison with normal rats.

In Figure 4, various detection methods are listed for observing the coffee-ring effect. One can check the nanoparticle deposition from the reflected light on the surface of the substrates with the naked eye. A miniature microscope in combination with a smartphone was utilized to enhance the sensitivity and convenience of observation of the coffee-ring effect. The data were recorded by the smartphone as images and uploaded to cloud terminals [27]. This system can be used as a portable device for point-of-care diagnostics. Together with fluorescent microscopy, we can further utilize thioflavin T (ThT) dye to stain the AuNP depositions to evaluate the fibrillation level of the Aβ, which was found to be an essential factor in diagnosing Alzheimer’s disease. ThT is known as a fluorescent dye that can specifically bind to Aβ fibrils^15^. The quantum yield of ThT molecules is increased after binding to the β-sheet sites of Aβ fibrils. The “turn-on” effect of ThT offers a fluorescent channel to selectively detect Aβ fibrils. The concentration of Aβ monomers is obtained from coffee-ring channel in the bright field, while the one of Aβ fibril is calculated from the fluorescent channel in the dark field of fluorescent microscopy. The Aβ fibrillation level is further estimated from the obtained Aβ fibril and monomer concentrations.

Aβ has been shown to be associated with Alzheimer’s disease. The Aβ biomarker is complex and includes various forms, such as monomers, oligomers, and mature fibrils [28]. Neuritic plaques consisting of aggregated Aβ in postmortem examinations are the most important marker for identifying Alzheimer’s disease. Soluble Aβ oligomers, which have high neural toxicity, are thought to be the main cause of neurologic damage in the development of Alzheimer’s disease [29]. A correlation between the concentration of Aβ monomers and Alzheimer’s disease has been suggested by a number of studies. However, some researchers believe that Aβ has physiological activities such as serving as a protector against oxidative stress. Overall, the physiological functions of Aβ in normal brain tissues and the development of Alzheimer’s disease are not well understood.

Alzheimer’s disease is associated with many other conditions, including diabetes [30], severe brain injury [31], brain ischaemia [32], uraemia, and hepatitis [33]. In most cases, the association between these diseases and Alzheimer’s disease is unclear. For example, in brain ischaemia, the mechanism by which ischaemia induces Alzheimer’s disease and the role of Aβ in the process have yet to be elucidated. These areas are of significant importance for revealing the pathogenesis of Alzheimer’s disease and for developing a potential cure for Alzheimer’s disease.

To study the association between brain ischaemic injury [34] and Alzheimer’s disease, surgeries were carried out using Sprague Dawley (SD) rat models. Long-term and short-term ischaemia were induced in SD rats. The Aβ monomer concentrations, fibril concentrations, and fibrillation levels of the cerebrospinal fluids were determined by the smart coffee-ring-effect-based optical platform (Figure 4c). The concentration of the Aβ monomer markedly increased in short-term brain ischaemia but decreased from this level in long-term ischaemia. However, the Aβ fibril concentration slightly increased in short-term brain ischaemia and then rapidly increased as the brain ischaemia progressed. Thus, we suggest that the Aβ fibrillation level in cerebrospinal fluids is a significant factor in long-term brain ischaemia. We further assume that Aβ may play a protective role against the development of brain ischaemia. We postulate that during the primary stages of brain ischaemia, the Aβ concentration increases. Then, this Aβ may start to aggregate when it reaches high concentrations, and the Aβ oligomers and fibrils that are formed accelerate the aggregation process. During the final stage, Aβ exists predominantly in oligomer and fibril forms, and the low concentration of the Aβ monomer is restored. These findings may help researchers understand the role of Aβ monomers and fibrils in brain tissues and the connection between brain ischaemia and Alzheimer’s disease.

## 4. Conclusions

The potential use of the coffee-ring effect in optical sensors has long been underestimated. Coffee-ring-effect-based optical sensors exhibit better sensitivity and more rapid throughout than conventional aggregation-induced colorimetric detection methods. The sensitivity of the coffee-ring-effect-based assay is improved by two to three orders of magnitude than conventional AuNP colorimetric methods. The assays also offer several additional advantages, including the extremely low sample volumes that are required, ultrahigh throughput, portability and point-of-care use, and compatibility with cloud computing. In this work, we not only offer a smart sensor for simultaneous Aβ monomer quantitation and evaluation of Aβ fibrillation level in cerebrospinal fluids, but also introduce a general sensing strategy appropriate for various analytical applications. As colloidal AuNP-based colorimetric sensors have become a well-studied field in the past decade, coffee-ring-effect-based optical sensors seem to be promising candidates to replace conventional noble-metal nanoparticle-based SPR colorimetric probes.

## Data Availability

The data presented in this study are available on request from the corresponding author. The data are not publicly available due to the restriction of the college.

## Supplementary Materials

The following supporting information can be downloaded at: https://www.mdpi.com/article/10.3390/biomimetics8080581/s1. Figure S1: The model of Monto Carlo simulations. Figure S2: Simulated radial distribution of monodispersed AuNPs (a) and trimers (b). Figure S3: Kinetics curve (a), selectivity (b), titration curve (c) and linear range of colorimetric sensor of Gel-AuNP for Aβ. Figure S4: TEM image (left) and HR-TEM image (right) of Gelsolin-modified AuNPs. Table S1: Comparison of coffee-ring effect assay with conventional colorimetric method using AuNPs.
