# Supplementary material for: Biomimetic Surface Engineering to Modulate the Coffee-Ring Effect for Amyloid-β Detection in Rat Brains
Source: Biomimetics (Basel). 2023 Dec 1;8(8):581. doi: 10.3390/biomimetics8080581 (PMC10742163; doi:10.3390/biomimetics8080581)
Supplement: Supplementary file 1 [file biomimetics-08-00581-s001.zip › biomimetics-2735390-supplementary.pdf]

biomimetics

Article

Biomimetic Surface Engineering to Modulate the Coffee-Ring Effect for Amyloid-β Detection in Rat Brains

Changxin Wang, Lei Li, Jiaze Li, Jun Zhang and Zhi-Bei Qu *

Department of Medicinal Chemistry, School of Pharmacy, Fudan University, 826 Zhangheng Road, Shanghai 201203, China; 23211030090@m.fudan.edu.cn (C.W.); 21301030041@m.fudan.edu.cn (L.L.)
* Correspondence: quzhibei@fudan.edu.cn

Chemicals and Reagents.

Gelsolin was purchased from Shanghai QCbio Science & Technologies Co., Ltd. Purified synthetic β-amyloid peptide (Aβ(1-42)) was purchased from ChinaPeptides Co,. Ltd (Shanghai, China). DNA was obtained from Sangon Company (Shanghai, China). $HAuCl_4$ and other chemicals were provided by Sinopharm Group Chemical Regent Co., Ltd. (Shanghai, China). The Aβ monomer, oligomers and fibrils were prepared as previously reported[1]. And all the other chemicals were used without further treatment unless men-tioned.

Instruments and Characterizations.

A JEM-2010 transmission electron microscope (JEOL Ltd. Japan) and BioScope atomic force microscope (NanoScope IIIa SPM System, Digital Instruments, Inc., U.S.A) were used to study the morphology. The UV-vis absorption spectra were recorded with a Shimadzu UV-1800 spectrophotometer (Tokyo, Japan). A Leika fluorescent microscopy was utilized to obtain optical and fluorescent images. An Android smartphone combined with a minimized microscopic lens was employed to construct a portable sensing plat-form.

Computational Simulations.

The Monto Carlo simulations were performed in a Python program. Briefly, the sim-ulations were based on the following hypothesises (Figure S1). 1. The outline of the drop-let is a sphere. 2. As the liquid evaporates, the radius of the droplet increases. 3. The na-nopartilces move randomly in the liquid. 4. When the nanoparticles move to the three-phase (liquid-air-solid) interface, the nanoparticles are deposited. 5. The simulation stops when the droplet is completely dried.

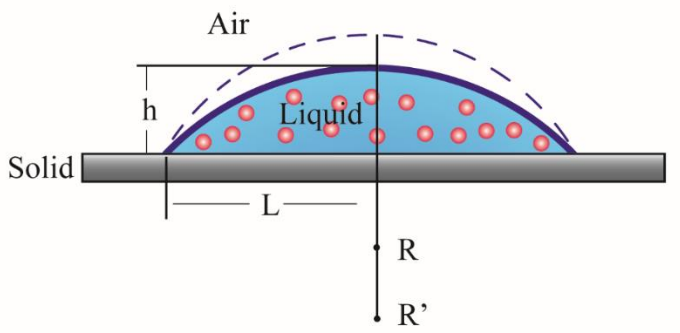

Figure S1. The model of Monto Carlo simulations.

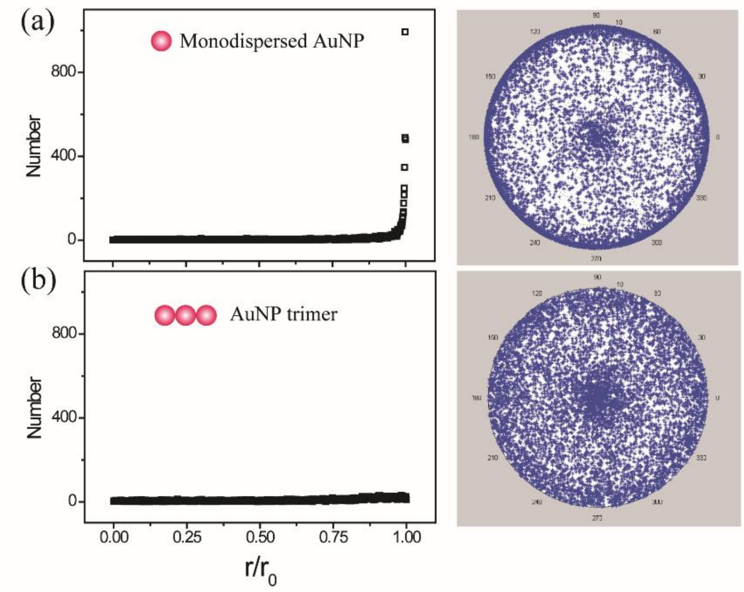

Figure S2. Simulated radial distribution of monodispersed AuNPs (a) and trimers (b).

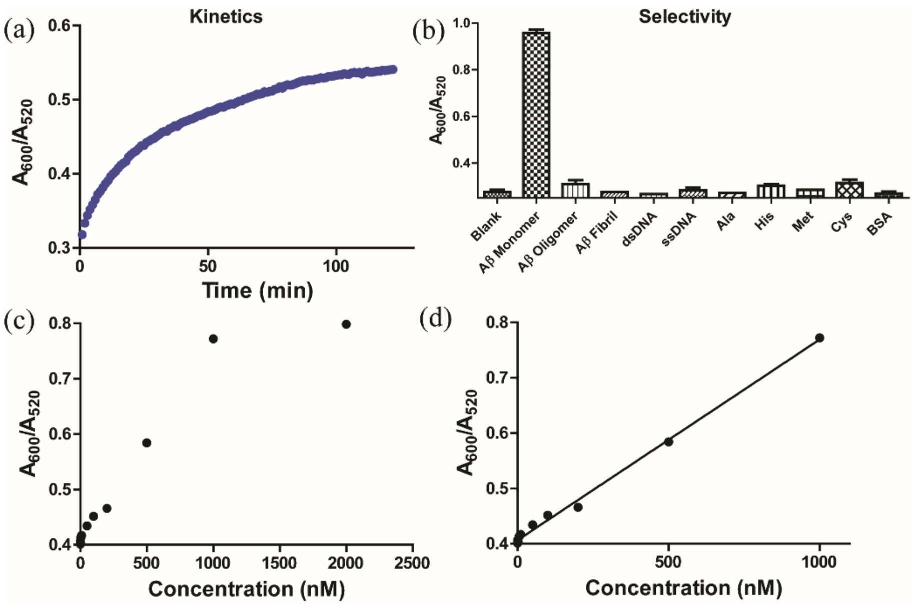

Figure S3. Kinetics curve (a), selectivity (b), titration curve (c) and linear range of colorimetric sensor of Gel-AuNP for Aβ.

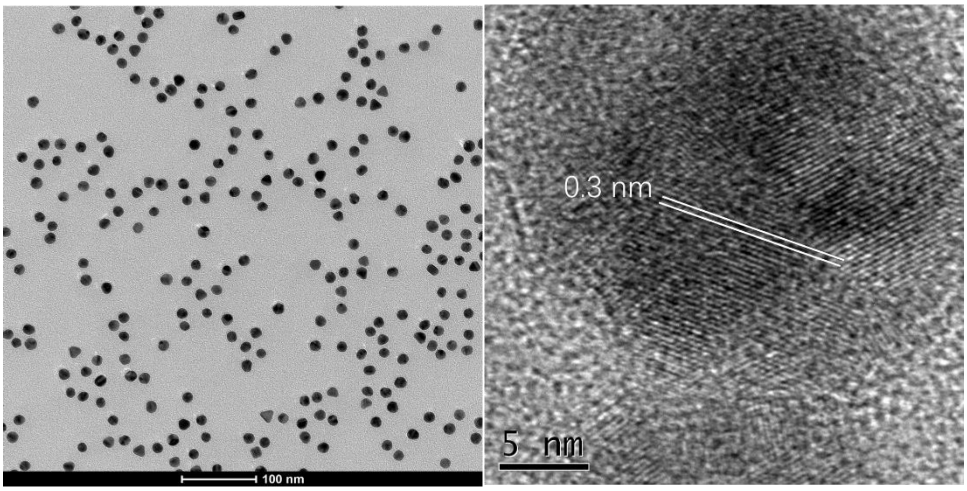

Figure S4. TEM image (left) and HR-TEM image (right) of Gelsolin-modified AuNPs.

Table S1. Comparison of coffee-ring effect assay with conventional colorimetric method using AuNPs.

	**Colorimetric assay**	**Coffee-ring Assay**
Volume of Sample of Use	1-2 mL	10-50 uL
Time of Measurement	5-15 min	~10 min
Sensitivity	0.5 nM	20 pM
Reproducibility	Good	Fair
Selectivity	Good	Good
Cost	High	Medium

[1] Zhang, X.;Tian, Y.; Li, Z.; Tian, X.; Sun, H.; Liu, H.; Moore, A.; Ran, C. Design and synthesis of curcumin analogues for in vivo fluorescence imaging and inhibiting copper-induced cross-linking of amyloid beta species in Alzheimer's disease. *J. Am. Chem. Soc.* **2013**, *135*, 16397–16409. https://doi.org/10.1021/ja405239v.
